# On the Utility of Administering Opium by the Urethra

**Published:** 1853-08

**Authors:** 


From the Brit. and For. Medico-Chirurgical Review.

*On the Utility of administering Opium by the Urethra.*

PRIOR to 1830, the author had published a statement of the great utility derivable from the introduction of opium into the urethra, to alleviate or overcome certain painful or obstinate affections, and especially stangulated hernia, violent colics, and some forms of ischuria. Since that period, the opportunities of a large hospital have furnished him with abundant means of confirming his statements. The dose employed has varied from two grains to six, the effect being the same whether the opium is introduced merely into the urethra or reaches the bladder. Narcotism is much more easily induced in this way in the male than in the female. It has been found very useful in strangulated inguinal hernia, the hernia sometimes returning of itself when the narcotism has been induced, but the aid of the taxis being also required in most cases. When the accompanying phlogosis has already led to certain results, such as adhesion, purulent effusion, &c., or where strangulation has supervened upon long-continued irreducible hernia, the operation will still be required; but even in these cases, the patients who have been subjected to the narcotism are much less sensible to the pain of the operation, and suffer much less from the subsequent traumatic fever. It is surprising to see in these patients, twenty or thirty minutes after the introduction of the opium, so complete a cessation of pains which had been so violent; the pulse becoming softer, the hernia less tense, and the whole system falling into a state of relaxation as a consequence of the narcotism. In two cases the excessive suffering from nephritic colic, consequent on the passage of calculi, was relieved, after all other means had been tried in vain. In six cases of retention of urine, in four of which catherism had become impossible, relief was obtained. In two cases of Neuralgia of the urethra from onanism, the same result followed the reiterated production of narcotism. In cancer of the uterus, the practice proves a highly useful means of palliating suffering. In spasmodic colic the method acts like a charm; and even in the inflammatory colic it is a powerful adjuvant to antiphlogistic treatment. This mode of introducing opium is also valuable in cases of painful organic disease of the alimentary canal, in which it is obstinately rejected by the mouth. Thus introduced, it does not cause the obstinate constipation or irritation of the digestive organs, nor the excessive narcotism, which large doses, often repeated, are apt to induce when administered by the ordinary modes.

